# Supplementary material for: Characteristics and clinical outcome in 312 patients with moderate to severe pneumonia due to SARS-COV-2 and hyperinflammation treated with anakinra and corticosteroids: A retrospective cohort study
Source: PLoS One. 2023 Mar 24;18(3):e0283529. doi: 10.1371/journal.pone.0283529 (PMC10038301; doi:10.1371/journal.pone.0283529)
Supplement: S1 File — (PDF) [file pone.0283529.s001.pdf]

S1 Table. Statistical analysis of all assessed variables in our cohort and among different treatment groups.

Cualitative Variables	Overall (n=312)	Group I (n=52)	Group II (n=153)	Group III (n=107)	Groups I/II	Groups I/III	Groups II/III
	n (%)	n (%)	n (%)	n (%)	p-value<br>OR (IC)	p-value<br>OR (IC)	p-value<br>OR (IC)
**Age (≥65)**	185 (59.3)	23 (44.2)	87 (56.9)	75 (70.1)	0.115<br>0.602 (0.319; 1.114)	0.002**<br>0.338 (0.170; 0.672)	0.030*<br>0.562 (0.333; 0.949)
**Sex (Male)**	204 (65.4)	31 (59.6)	103 (67.3)	70 (65.4)	0.313<br>0.717 (0.375; 1.371)	0.476<br>0.780 (0.394; 1.544)	0.749<br>1.089 (0.646; 1.836)
**Comorbidities**							
HTA	181 (58)	30 (57.7)	86 (56.2)	65 (60.7)	0.852<br>1.062 (0.562; 2.007)	0.712<br>0.881 (0.449; 1.727)	0.465<br>0.829 (0.502; 1.371)
EPOC/Asthma	67 (21.5)	9 (17.3)	29 (19.0)	29 (27.1)	0.792<br>0.895 (0.392; 2.041)	0.174<br>0.563 (0.244; 1.298)	0.120<br>0.629 (0.350; 1.132)
Obesity	124 (39.7)	24 (46.2)	59 (38.6)	41 (38.3)	0.335<br>1.366 (0.724; 2.577)	0.346<br>1.380 (0.706; 2.697)	0.968<br>1.010 (0.608; 1.679)
Ischemic cardiology	26 (8.3)	4 (7.7)	14 (9.2)	8 (7.5)	0.748<br>0.827 (0.260; 2.636)	0.961<br>1.031 (0.296; 3.595)	0.633<br>1.246 (0.504; 3.084)
Heart failure	39 (12.5)	6 (11.5)	14 (9.2)	19 (17.8)	0.616<br>1.295 (0.470; 3.566)	0.312<br>0.604 (0.226; 1.617)	0.040*<br>0.466 (0.223; 0.978)
Diabetes	98 (31.4)	14 (26.9)	44 (28.8)	40 (37.4)	0.800<br>0.913 (0.451; 1.848)	0.191<br>0.617 (0.298; 1.277)	0.143<br>0.676 (0.400; 1.143)
Renal insufficiency	43 (13.8)	7 (13.5)	16 (10.5)	20 (18.7)	0.553<br>1.332 (0.515; 3.444)	0.410<br>0.667 (0.266; 1.720)	0.059<br>0.508 (0.250; 1.034)
Polypathological	35 (11.2)	6 (11.5)	15 (9.8)	14 (13.1)	0.722<br>1.200 (0.440; 3.274)	0.783<br>0.866 (0.313; 2.402)	0.408<br>0.722 (0.333; 1.566)
**Treatments**							
Corticosteroids	300 (96,5)	42 (80,8)	152 (99,3)	106 (100)	0,000**<br>0.232 (0.086; 0.624)	0,404<br>0.747 (0.313; 1.784)	0,000**<br>3.222 (1.325; 7.835)
Remdesivir	43 (13,8)	6 (11,5)	25 (16,3)	12 (11,2)	0,404<br>0.668 (0.258; 1.731)	0,244<br>1.033 (0.365; 2.925)	0,952<br>1.546 (0.739; 3.233)
Tocilizumab	23 (7,4)	0 (0)	7 (4,6)	16 (15)	0,117<br>NA	0,004**<br>1.571 (1.388; 1.779)	0,003**<br>0.273 (0.108; 0.688)
Heparin	311 (99,7)	52 (100)	153 (100)	106 (99,1)	NA<br>NA	0,857<br>NA	1,000<br>NA
**Vaccination**	35 (11.2)	11 (21.2)	14 (9.2)	10 (9.3)	0,022*<br>0,375 (0,158; 0,890)	0,957<br>0,384 (0,151; 0,975)	0,039*<br>1,024 (0,437; 2,399)
**TAC**	109 (35.3)	16 (30.8)	60 (39.7)	33 (31.1)	0.249<br>0.674 (0.344; 1.321)	0.963<br>0.983 (0.479; 2.016)	0.158<br>1.495 (0.863; 2.465)
**CORADS 5**	49 (15.7)	11 (21.2)	23 (15.0)	15 (14.0)	0.305<br>1.516 (0.682; 3.374)	0.820<br>1.646 (0.696; 3.891)	0.254<br>1.085 (0.537; 2.192)
**Serology month (positive)**	202 (97.1)	35 (100)	92 (96.8)	75 (96.2)	0.287<br>NA	0.587<br>NA	1.000<br>1.227 (0.241; 6.255)
**Thrombosis**	21 (6.7)	3 (5.8)	12 (7.8)	6 (5.6)	0.620<br>0.719 (0.195; 2.656)	1.000<br>1.031 (0.247; 4.295)	0.485<br>1.433 (0.520; 3.944)
**Previous ACO**	31 (9.9)	3 (5.8)	11 (7.2)	17 (15.9)	0.726<br>0.790 (0.212; 2.951)	0.071<br>0.324 (0.091; 1.161)	0.026*<br>0.410 (0.184; 0.916)
**Heparin at discharge**	181 (58)	31 (67.4)	91 (63.6)	59 (60.2)	0.643<br>1.181 (0.584; 2.389)	0.406<br>1.366 (0.654; 2.856)	0.589<br>1.157 (0.682; 1.963)
**Evolution 30 days**							
Medical discharge	201 (64.4)	38 (73.1)	103 (67.3)	60 (56.6)	0.439<br>1.389 (0.654; 2.653)	0.039<br>2.126 (1.033; 4.377)	0.065<br>1.614 (0.969; 2.687)
Exitus	81 (26.0)	1 (1.9)	3 (2.0)	1 (0.9)	0.419<br>0.733 (0.344; 1.560)	0.417<br>0.722 (0.327; 1.591)	0.956<br>0.985 (0.564; 1.718)
UCI	4 (1.3)	11 (21.2)	41 (26.8)	29 (27.4)	1.000<br>0.980 (0.100; 9.637)	0.712<br>NA	0.386<br>1.713 (1.545; 1.900)

Remains hospitalized	25 (8.0)	2 (3.8)	6 (3.9)	17 (16.0)	1.000<br>(0.980; 5.013)	0.028*<br>0.212 (0.047; 0.954)	0.001**<br>0.216 (0.082; 0.568)

OR (CI)): Odd Ratio and Confidence Interval for the OR,

NA: Not applicable due to the presence of null frequencies ** p-value under 0.01 * p-value under 0.05

**Quantitative variables**	**Overall**<br>**(n=312)**	**Group I**<br>**(n=52)**	**Group II**<br>**(n=153)**	**Group III (n=107)**	**Groups I-II**	**Groups I-III**	**Groups II-III**
	**Mean (SD)**	**Mean (SD)**	**Mean (SD)**	**Mean (SD)**	**p-value (CI)**	**p-value (CI)**	**p-value (CI)**
**Days of…**							
Symptoms before Anakinra	10.96<br>(3.94)	8,88<br>(3.46)	10.33<br>(3.26)	12.90<br>(4.26)	0,007**<br>(-2.502; -0.391)	0.000**<br>(-5.367; -2.659)	0.000**<br>(-3.538; -1.595)
UCI	12.63<br>(15.98)	9.50<br>(10.08)	17.38<br>(20.78)	6.92<br>(4.32)	0.166<br>(-19.219; 3.457)	0.429<br>(-4.099; 9.265)	0.036*<br>(0.733; 20.195)
Intubation	15.09<br>(16.96)	12.25<br>(9.91)	17.73<br>(20.02)	8.00<br>(5.29)	0.608<br>(-27.608; 16.642)	0.478<br>(-9.497; 17.997)	0.358<br>(-11.993; 31.460)
Hospitalization	17.04<br>(11.73)	11.82<br>(8.24)	15.65<br>(12.13)	17<br>(14-24)	0.002**<br>(-7.432; -0.224)	0.000**<br>(-12.779; -6.563)	0.000**<br>(-8.767; -2.940)
Treatment with Anakinra	13.42<br>(13.83)	11.02<br>(8.18)	13.54<br>(14.45)	14.39<br>(14.98)	0.239<br>(-6.723;1.684)	0.135<br>(-7.807; 1.060))	0.645<br>(-4.500; 2.793)
Heparin at discharge	11.70<br>(15.74)	14.07<br>(15.68)	11.86<br>(19.33)	22.32<br>(16.04)	0.592<br>(-5.958; 10.385)	0.175<br>(-2.012; 10.502)	0.506<br>(-3.992; 8.055)
**Anakinra dose**	985.90<br>(563.23)	925.00<br>(610.65)	977.78<br>(551.94)	1027.10<br>(557.55)	0.563<br>(-232.316; 126.760)	0.295<br>(-294.206; 90.001)	0.481<br>(-186.870; 88.220)
**PROFUND**	4.6<br>(2.91)	3.33<br>(2.81)	5.00<br>(3.14)	4.71<br>(2.76)	0.273<br>(-4.779; 1.445)	0.321<br>(-4.221; 1.460)	0.797<br>(-1.963; 2.534)
**Data at admission**							
SaFi	399.19<br>(81.66)	377.40<br>(85.37)	400.02<br>(81.79)	408.60<br>(78.37)	0.090<br>(-48.791; 3.559)	0.024*<br>(-58.144; -4.244)	0.398<br>(-28.531; 11.374)
SaFi at the beginning of Anakinra treatment	228.60<br>(104.48)	271.73<br>(114.30)	224.78<br>101.05)	213.09<br>(99.62)	0.006**<br>(13.866; 80.040)	0.001**<br>(23.706; 93.568)	0.357<br>(-13.248; 36.616)
PCR (mg/L)	105.75<br>(75.66)	140.83<br>(82.93)	109.16<br>(70.60)	83.41<br>(71.87)	0.009**<br>(8.097; 55.235)	0.000**<br>(32.016; 82.821)	0,005**<br>(7.777; 43.728)
Ferritin (µg/L)	1023.33<br>(837.68)	1136.12<br>(1006.46)	1020<br>(824.47)	971.30<br>(763.52)	0.413<br>(-162.937; 394.716)	0.256<br>(-120.94; 450.57)	0.634<br>(-153.182; 251.038)
D-Dimer (mg/L)	6.09<br>(52.59)	1.73<br>(4.76)	8.56<br>(73.70)	4.82<br>(20.17)	0.506<br>(-27.032; 13.372)	0.279<br>(-8.698; 2.524)	0.614<br>(-10.872; 18.358)
Lymphocytes (x10^9)	1084.32<br>(2881.35)	869.81<br>(398.19)	989,93<br>(99.36)	1322.27<br>(4747.90)	0.401<br>(-401.545; 161.299)	0.494<br>(-1757.53; 852.61)	0.415<br>(-1134.63; 469.95)
SCOPE	6.51<br>(1.90)	6.9<br>(1.92)	6.44<br>(1.84)	6.41<br>(1.96)	0.123<br>(-0.127; 1.058)	0.141<br>(-0.164; 1.145)	0.918<br>(-0.453; 0.503)
**Data at 72 hours**							
PCR (mg/L)	43.49<br>(56.16)	47.39<br>(50.65)	43.22<br>(55.69)	41.84<br>(59.89)	0.637<br>(-13.213; 21.541)	0.569<br>(-13.679; 24.774)	0.854<br>(-13.369; 16.136)
Ferritin (µg/L)	1005.18<br>(841.78)	1036.71<br>(903.64)	992.61<br>(843.07)	1006.63<br>(814.53)	0.752<br>(-230.707; 318.912)	0.836<br>(-255.674; 315;837)	0.897<br>(-227.829; 199.787)
D-Dimer (mg/L))	6.30<br>(48.31)	3.13<br>(7.02)	9.09<br>(68.63)	3.98<br>(7.61)	0.533<br>(-24.799; 12.874)	0.551<br>(-3.278; 1.757)	0.458<br>(-8.591; 18.996)
Lynmphocytes (x10^9)	1334<br>(1394.34)	1298.12<br>(705.96)	1391.42<br>(1260.65)	916.93<br>(1794.73)	0.614<br>(-457.894; 271.288)	0.923<br>(-486.737; 536.888)	0.548<br>(-269.239; 505.996)
**Data at 30 days**							
PCR (mg/L)	28.52<br>(40.37)	44.857<br>(55.59)	19.48<br>(31.89)	31.08<br>(40.87)	0.177<br>(-26.688; 77.449)	0.543<br>(-32.985; 60.533)	0.406<br>(-39.837;16.624
Ferritin (µg/L)	509.06<br>(725.14)	762.57<br>(342.83)	545.56<br>(1074.0)	378.02<br>(363.45)	0.610<br>(-655.669; 1089.687)	0.024*<br>(54.503; 714.600)	0.537<br>(-379.647; 714.552)

SD: Standard deviation

CI: Confidence interval for the mean difference and p-value based on T-test (normalization is assumed based on the sample size.

NA: Not Applicable   ** p-value under 0.01   * p-value under 0.05
